# The Role of Natural Flavonoid Quercetin in Combating Tet(X3)/Tet(X4)-Positive Bacterial Infections

**DOI:** 10.3390/microorganisms14081692

**Published:** 2026-08-01

**Authors:** Xiaokun Zhan, Lifeng Zhou, Hanyu Wang, Lihao Wang, Lizhi Tian, Yonglin Zhou, Kuan Gu, Houru Liu

**Affiliations:** 1 Institute of Microbe and Host Health, College of Agriculture and Forestry, Linyi University, Linyi 276000, China; 202326210203@lyu.edu.cn (X.Z.); 202526210107@lyu.edu.cn (H.W.); 202326210138@lyu.edu.cn (L.W.); 2 Key Laboratory of Ministry of Education for Conservation and Utilization of Special Biological Resources in the Western China, School of Life Sciences, Ningxia University, Yinchuan 750021, China; 12025131024@stu.nxu.edu.cn (L.Z.); 18609507142@163.com (L.T.); zhouyonglin333@nxu.edu.cn (Y.Z.); 3 School of Chinese Medicine, Nanjing University of Chinese Medicine, Nanjing 210023, China

**Keywords:** quercetin, tet(X3)/tet(X4), tetracycline, inhibitor, drug resistance

## Abstract

The increased occurrence of tetracycline resistance (conferred by the *tet*(X3)/*tet*(X4) resistance genes) poses a major challenge to the clinical treatment of infections using tetracyclines, including tigecycline, which is the “last-resort” therapeutic agent. Thus, there is an urgent need to develop inhibitors targeting Tet(X3)/Tet(X4) to address the crisis of tetracycline resistance. This study revealed that quercetin is an inhibitor of Tet(X3)/Tet(X4), which, when combined with different tetracyclines, can exert synergistic growth-inhibitory effects against Tet(X3)/Tet(X4)-positive bacteria (fractional inhibitory concentration < 0.5). Quercetin + tigecycline significantly decreased the biofilm-forming ability of bacteria. Mechanistic investigations revealed that quercetin inhibits the catalytic activity of Tet(X4) against tetracycline antibiotics by binding to the catalytic active site. In the *Galleria mellonella* larval infection model of Tet(X4)-positive *E. coli* J53p47EC or Tet(X4)-negative *E. coli* J53, tigecycline + quercetin enhanced the survival rate, decreased melanization, and suppressed bacterial load. Thus, the findings of this study will enable the development of novel Tet(X3)/Tet(X4)-targeted therapeutic strategies for clinical infections caused by drug-resistant *Enterobacteriaceae* and *Acinetobacter baumannii*, especially Tet(X3)/Tet(X4)-positive pathogens.

## 1. Introduction

Irrational usage of antimicrobial agents has contributed to the emergence and dissemination of antimicrobial resistance [1,2]. According to the World Bank Group, antimicrobial resistance is projected to contribute to more than 10 million deaths annually worldwide by 2050 and increase healthcare expenditures to hundreds of billions of U.S. dollars if adequate containment measures are not implemented [3]. Furthermore, antibiotic-resistant infections can decrease the production rate of the livestock farming industry by up to 7.5% [3]. Among the Gram-negative pathogens, *Escherichia coli*, *Klebsiella pneumoniae*, and *Acinetobacter baumannii* are associated with the highest prevalence and most severe resistance profiles [4].

Tetracyclines, which exhibit broad-spectrum bacteriostatic activities, are extensively utilized antimicrobials in both human and veterinary settings [5]. Tigecycline, a glycylcycline derivative of tetracycline, serves as the “last-resort” antibiotic for the treatment of complicated intra-abdominal infections, skin and soft tissue infections, and other infections caused by extensively drug-resistant (XDR) Gram-negative pathogens harboring resistance enzymes, such as New Delhi metallo-β-lactamase-1 (NDM-1) and *Klebsiella pneumoniae* carbapenemase-2 [6,7,8]. Thus, regulatory authorities of multiple countries have prohibited the use of tigecycline in food-producing animals to mitigate the risk of resistance selection and cross-species transmission.

Tetracycline resistance in pathogenic bacteria has been attributed to the following two well-characterized mechanisms [9,10,11]: tetracycline-specific efflux pumps, such as Tet(A) and Tet(K) [9], and ribosomal protection proteins, including Tet(M) and Tet(O) [9,10]. These mechanisms typically confer low-level resistance to tetracyclines. In 2019, Chinese scientists identified two plasmid-encoded tetracycline destructases, namely Tet(X4) and Tet(X3), in animal-derived pathogenic bacteria. Tet(X3) and Tet(X4) confer high-level resistance to all tetracyclines, including tigecycline and the new tetracyclines (omadacycline and eravacycline) approved by the Food and Drug Administration [12]. The Tet(X) variants exhibit sequence homology. For example, Tet(X3) and Tet(X4) share 87.07% nucleotide sequence identity [12,13,14]. Tet(X3) and Tet(X4) function as flavin adenine dinucleotide (FAD)-dependent monooxygenases, catalyzing the oxidative degradation of tetracyclines in vitro in the presence of both nicotinamide adenine dinucleotide phosphate (NADPH) and O_2_ [14].

Tet(X3) and Tet(X4), which are multidrug-resistant determinants, facilitate both horizontal and vertical transmission among prevalent Gram-negative pathogens, including *E. coli*, *A. baumannii*, and *K. pneumoniae*, amplifying the risk of tetracycline resistance dissemination [15,16,17]. *Acinetobacter* strains of bovine origin co-harbor Tet(X3) and NDM-1. Thus, Tet(X3) and Tet(X4) can coexist with distinct resistance determinants within a single bacterial isolate, contributing to multidrug-resistant or even pan-drug-resistant phenotypes [18]. Tetracyclines are crucial for controlling drug-resistant bacteria. The molecular mechanism of Tet(X3)/Tet(X4)-mediated resistance has previously been elucidated. The development of strategies to screen Tet(X3)/Tet(X4) inhibitors is a critical frontier in antimicrobial research to combat resistant infections [19,20,21]. However, the challenges associated with the development of Tet(X) inhibitors include poor drug-likeness, insufficient target selectivity, lack of in vivo systematic investigations, lagging clinical translation, and uncharacterized risks of inhibitor-mediated resistance. Consequently, Tet(X) inhibitors have not been evaluated in clinical trials. Thus, there is a need for developing effective interventions against Tet(X)-mediated tetracycline resistance.

This study aimed to screen natural flavonoids for effective Tet(X3)/Tet(X4) enzymatic inhibitors, verify the in vitro synergistic antibacterial and anti-biofilm activities of candidate compounds when used in combination with tetracyclines, clarify the molecular binding mechanism between the candidate compound and Tet(X4) via molecular docking, molecular dynamics (MD) simulation, and site-directed mutagenesis, and validate the in vivo therapeutic potential of the combination regimen in *Galleria mellonella* larval infection models. The findings of this study provide an adjuvant strategy using a natural compound to restore the clinical efficacy of tigecycline against Tet(X3)/Tet(X4)-mediated pan-tetracycline resistance.

## 2. Materials and Methods

### 2.1. Cell Lines and Cell Culture

Human lung carcinoma (A549) and mouse mononuclear macrophage (J774 A.1) cells obtained from the American Type Culture Collection were cultured in high-glucose Dulbecco’s modified Eagle’s medium (Gibco, New York, NY, USA) supplemented with 10% fetal bovine serum and 1% (*w*:*v*) penicillin–streptomycin at 5% CO_2_ and 37 °C.

### 2.2. Bacterial Strains and Reagents

The characteristics of all bacterial strains, including Tet(X3)/Tet(X4)-positive/negative clinical isolates and Tet(X3)/Tet(X4)-positive/negative laboratory strains, have been detailed in our previous studies [19,21,22]. The strains were stored in a −80 °C refrigerator, used, and identified according to standard microbiological procedures (www.atcc.org). In this study, bacterial culture and antimicrobial susceptibility tests were performed using Luria–Bertani broth and Mueller–Hinton broth (MHB), respectively. Quercetin powder was dissolved in dimethyl sulfoxide (DMSO) to prepare a 20 mg/mL stock solution, which was aliquoted and stored at −20 °C in the dark. The stock solution was diluted to the required working concentrations for the experiments. The final volume of DMSO in all treatment groups was maintained below 0.1%. Tetracycline antibiotics and quercetin were purchased from Shanghai Aladdin Biochemical Technology Co., Ltd., (Shanghai, China) and Shanghai Yuanye Bio-Technology Co., Ltd., (Shanghai, China) respectively. All other conventional chemical reagents were purchased from Sigma–Aldrich Inc. (St. Louis, MO, USA).

In this study, all microbiological experiments comprised the following three control groups: sterility control group, blank sterile medium; growth control group, untreated bacteria; solvent control group, equal volume of DMSO used to dissolve quercetin. The final DMSO concentration was ≤0.1% (*v*/*v*), which did not exhibit independent antibacterial activity.

### 2.3. Enzyme Inhibition Assay

The reaction mixture for the enzyme inhibition assay mainly comprised purified Tet(X3)/Tet(X4) protein (purity > 80% (for both); concentration: 2.35 mg/mL (Tet(X3)) and 2.71 mg/mL (Tet(X4))), quercetin (potential inhibitor of Tet(X3)/Tet(X4)), NADPH, Mg^2+^, and O_2_. The optical density at 400 nm (OD_400nm_) of the reaction mixture was determined at 37 °C for 60 min to evaluate the inhibitory effect of quercetin on Tet(X3) or Tet(X4) [19]. The inhibitory effect of quercetin on the catalytic activity of Tet(X3)/Tet(X4) was determined by comparing the changes in absorbance values of each sample relative to the untreated control for 1 h.

### 2.4. Recombinant Tet(X3)/Tet(X4) Construction, Expression and Purification

The construction of the recombinant plasmids pGEX-4T-1-Tet(X3)/Tet(X4) and the specific mutants, the establishment of strains harboring the wild-type and mutant plasmids, and the expression and purification of Tet(X4) were performed as reported previously [19,22].

### 2.5. Antimicrobial Susceptibility Test

The minimum inhibitory concentrations (MICs) of tetracyclines (tigecycline, minocycline, methacycline, and tetracycline) and quercetin were determined using the checkerboard broth microdilution assay in MHB with a sterile 96-well microtiter plate, following the Clinical and Laboratory Standards Institute guidelines [23]. Briefly, serial twofold dilutions of tetracyclines and quercetin were prepared in cation-adjusted MHB and dispensed into a 96-well microtiter plate in an orthogonal matrix format. The concentrations of the compounds ranged from sub-inhibitory to supra-inhibitory levels. Each well was inoculated with a standardized bacterial suspension at a final inoculum density of approximately 5 × 10^5^ colony-forming units (CFUs) per milliliter. The samples were incubated under aerobic conditions at 37 °C for 16–24 h. The MIC values of the compounds against the tested bacterial strains were defined as the lowest concentrations of antimicrobial agents at which visible bacterial growth was not observed. The fractional inhibitory concentration (FIC) values were calculated based on the concentrations of the antibiotic and quercetin at their optimal inhibitory combination, as well as their respective MICs, as follows: FIC index (FICI) = MIC_Antibiotic in combination_/MIC_Antibiotic alone_ + MIC_Quercetin in combination_/MIC_Quercetin alone_. FICI ≤ 0.5 indicates the synergistic effects of antibiotics and quercetin.

### 2.6. Growth Curves

Overnight cultured bacteria were diluted and incubated at 37 °C with shaking until an OD_600nm_ value of 0.3 was achieved. Different concentrations of quercetin (final concentrations: 0, 8, 16, 32, 64 μg/mL) were transferred to five sterile Erlenmeyer flasks and mixed with the tested bacterial culture. The mixed bacterial systems were cultured at 37 °C with shaking for 5 h. The OD_600nm_ value was recorded once every 1 h and was used to generate the bacterial growth curves.

### 2.7. Time-Dependent Killing Assay

The OD_600nm_ value of the *E. coli* DH5α + pAM401-*tet*(X3) and *E. coli* DH5α + pAM401-*tet*(X4) cultures was allowed to reach 0.1. The bacterial cultures were incubated with different concentrations of quercetin, tigecycline, or the quercetin + tigecycline combination (5 × 10^5^ CFU/mL). The samples were then plated at the indicated time point (0–24 h). The CFUs on the solid agar plate in different treatment groups were calculated and used to prepare the sterilization curve.

### 2.8. Bacterial Viability Determination

The OD_600nm_ value of the *E. coli* DH5α + pAM401-*tet*(X3) and *E. coli* DH5α + pAM401-*tet*(X4) cultures was allowed to reach 0.1 (approximately 1 × 10^8^ CFU/mL). The bacterial cultures were incubated with different concentrations of quercetin, tigecycline, or the quercetin + tigecycline combination for 5 h. Next, the bacterial cells were collected, washed, and subjected to live/dead staining with SYTO 9 and propidium iodide (LIVE/DEAD BacLight Bacterial Viability Kit, Thermo Fisher Scientific, Wilmington, DE, USA), following the manufacturer’s instructions.

### 2.9. Biofilm Inhibition Assays

Overnight cultured *E. coli* 47EC or *A. baumannii* 34AB were diluted and cultured with shaking at 200 rpm and 37 °C until the logarithmic phase. The bacterial cultures were diluted with fresh broth in the presence or absence of quercetin, tigecycline, or the quercetin + tigecycline combination in a 24-well plate (the initial bacterial amount in each was 0.1 at OD_600nm_) and incubated for 24 h at 37 °C to allow biofilm formation. After removing the bacterial culture supernatant, the biofilm was stained with 0.1% crystal violet (CV). The biofilm was solubilized with 30% glacial acetic acid. The content of biofilm was quantified by measuring the OD_570nm_ values.

### 2.10. Scanning Electron Microscopy (SEM) Analysis

The morphology of *E. coli* DH5α+pAM401-*tet*(X4) treated with quercetin, tigecycline, or the quercetin + tigecycline combination was examined using SEM. Briefly, overnight cultured bacteria were diluted and cultured to the logarithmic growth phase. The bacteria were resuspended and diluted to a density of 1.0 × 10^8^ CFU/mL. After different treatments for 5 h, diluted bacteria were fixed in 2.5% glutaraldehyde. The bacterial samples were washed (sterile PBS buffer), dehydrated using ethanol (30%, 50%, 70%, 90%, and 100%, respectively), dried in a 55 °C incubator, overgilded, and observed under a scanning electron microscope (SU5000, HITACHI, Hitachi Construction Machinery Co., Ltd. Tokyo, Japan).

### 2.11. Resistance Development Analysis

The bacteria were treated with quercetin (64 μg/mL), tigecycline (1 μg/mL or 4 μg/mL), or quercetin (64 μg/mL) + tigecycline (1 μg/mL) for 29 days. From day 2, the MICs of the bacterial cultures were determined in a 96-well microtiter plate using the broth microdilution method once every two days [19].

### 2.12. Molecular Docking and MD Simulations

Gromacs 2024 was used for MD simulations of the docked protein–ligand complex under the following conditions: ff14SB parameter for protein; General Amber Force Field parameter for ligand; TIP3P water; periodic boundary conditions; a 4-step workflow. The PDB code (PDB ID:7EPV) of Tet(X4) was acquired from the Protein Data Bank.

All MD simulations were performed for 100 ns with a van der Waals interaction cutoff of 1.0 nm and electrostatic interaction handled via Particle Mesh Ewald. The simulation system underwent the following four sequential equilibration steps: energy minimization, NVT temperature equilibration, NPT pressure equilibration, and production simulation. Three parallel repeated simulation replicates were performed for the Tet(X4)-quercetin complex and the free Tet(X4) protein system.

To further calculate the interaction energy of the Tet(X4)-quercetin complexes (total contribution: E*_total_*; the van der Waals contribution: E*_vdw_*; electrostatic contribution: E*_ele_*; solvation contribution: E*_solv_*) [24], this study analyzed molecular mechanics/Poisson–Boltzmann surface area (MM-PBSA) using the Amber 10 package.

### 2.13. Cell Toxicity Assays

A549 cells and J774A.1 cells were incubated with different concentrations of quercetin (0, 4, 8, 16, 32, or 64 μg/mL) for 5 h. The cell viability was determined using a cytotoxicity detection kit (lactate dehydrogenase) (Roche, Basel, Switzerland), following the manufacturer’s instructions.

Fresh rabbit erythrocytes (Thermo Scientific^TM^ Heraeus^TM^ Pico^TM^/Fresco^TM^ 17/21) were washed at 37 °C and 1000× *g* for 5 min and incubated with different concentrations of quercetin (0, 4, 8, 16, 32, or 64 μg/mL) at 37 °C for 1 h. The samples were centrifuged at 37 °C and 1000× *g* for 5 min. The OD_570nm_ value of the supernatant was determined using a microplate reader (Tecan Group AG, Männedorf, Switzerland).

### 2.14. G. mellonella Larvae Infection Model

The Tet(X4)-positive *E. coli* J53p47EC or Tet(X4)-negative *E. coli* J53 cultures were microinjected through the left pro-leg to establish the infection model. At 2 h post-infection, the *G. mellonella* larvae were allotted into the following six groups: quercetin group, infected with *E. coli* J53p47EC and treated with quercetin (40 mg/kg body weight); tigecycline *E. coli* J53p47EC group, infected with *E. coli* J53p47EC and treated with tigecycline (8 mg/kg body weight); tigecycline *E. coli* J53 group, infected with *E. coli* J53 and treated with tigecycline (8 mg/kg body weight); quercetin + tigecycline group, infected with *E. coli* J53p47EC and treated with quercetin (40 mg/kg body weight) and tigecycline (8 mg/kg body weight); *E. coli* J53p47EC group, infected with *E. coli* J53p47EC; *E. coli* J53 group, infection with *E. coli* J53) [22]. The larval mortality was determined based on melanization and lack of response to mechanical stimulation. The therapeutic efficacy of the quercetin + tigecycline combination was monitored for 7 days [25,26].

A sublethal-dose infection model was also established to investigate bacterial clearance dynamics. At 48 h post-therapeutic intervention, the larvae were euthanized. The bacterial colonization and melanization in the larvae were determined [25,26].

### 2.15. Statistical Analysis

Each experiment was independently performed thrice to ensure reliability and reproducibility. Statistical analysis was performed using GraphPad Prism (version 9.5.0). The data are expressed as the mean ± standard deviation. Sample size is mentioned in the figure legends. Means were compared using the unpaired Student’s *t*-test or one-way analysis of variance (* *p* < 0.05; ** *p* < 0.01).

## 3. Results

In this section, the results of the in vitro, molecular, and in vivo experiments are presented. This study identified a natural flavonoid that inhibits Tet(X3)/Tet(X4) and elucidated its synergistic anti-resistance mechanisms.

### 3.1. Quercetin Is a Potential Inhibitor of Tet(X3)/Tet(X4)

**Figure 1:**
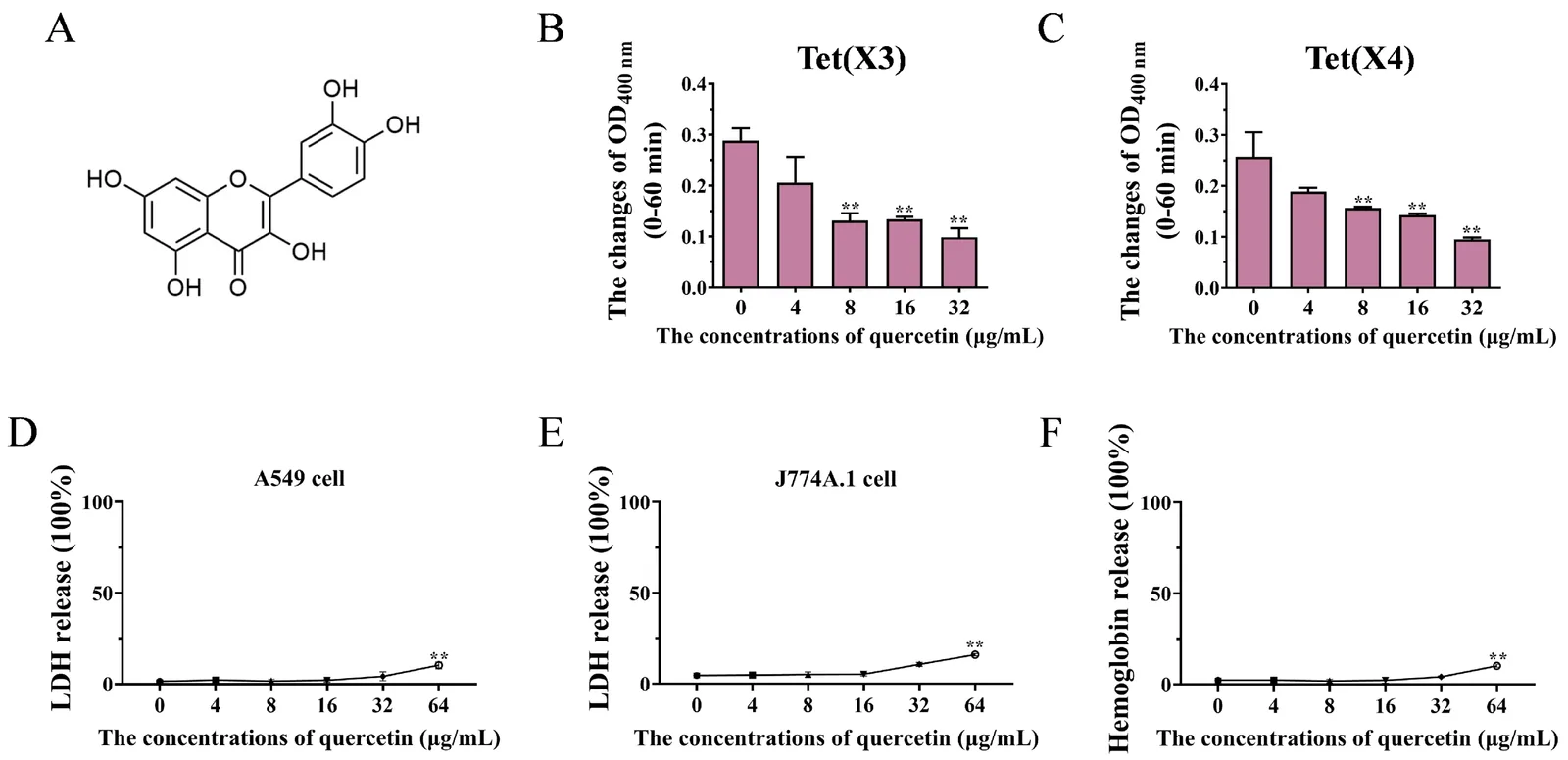
**Screening of potential inhibitors of Tet(X3)/Tet(X4).** (**A**) Chemical structure of quercetin. The catalytic activities of Tet(X3) (**B**) and Tet(X4) (**C**) in the presence of the indicated concentrations of quercetin were evaluated based on the optical density values at 400 nm (OD_400nm_) (0–60 min). Effect of different concentrations of quercetin on the viability of A549 (**D**) and J774A.1 (**E**) cell lines. (**F**) Effect of quercetin on the rabbit red blood cells. Experiments were independently performed thrice. Data are represented as mean ± standard deviation. ** *p* < 0.01. LDH, lactate dehydrogenase.

The pharmacological effects of several flavonoids have piqued the interest of the scientific community. Tet(X3) and Tet(X4), which are NADP-dependent oxidoreductases, hydrolyzed tetracycline, as evidenced by the changes in the OD_400nm_ values. Tetracycline degradation also resulted in color changes. Based on this biochemical activity, this study developed a method to screen the inhibitors of Tet(X3) or Tet(X4). Quercetin, a natural compound, was identified as a Tet(X3) or Tet(X4) inhibitor (Figure 1A). Additionally, quercetin exerted inhibitory activities against both Tet(X3) and Tet(X4) (Figure 1B,C). Cytotoxic assays are a prerequisite for conducting in vivo experiments. Therefore, this study examined the cytotoxic effects of quercetin against different cell lines. As shown in Figure 1D,E, quercetin exerted negligible cytotoxic effects against A549 cells and J774A.1 cells at the indicated effective concentrations. Additionally, quercetin (at concentrations of 4–64 μg/mL) did not exert significant hemolytic activities (Figure 1F).

### 3.2. Quercetin Enhances the Antibacterial Activity of Tetracyclines Against Tet(X3)/Tet(X4)-Positive Bacterial Strains

**Figure 2:**
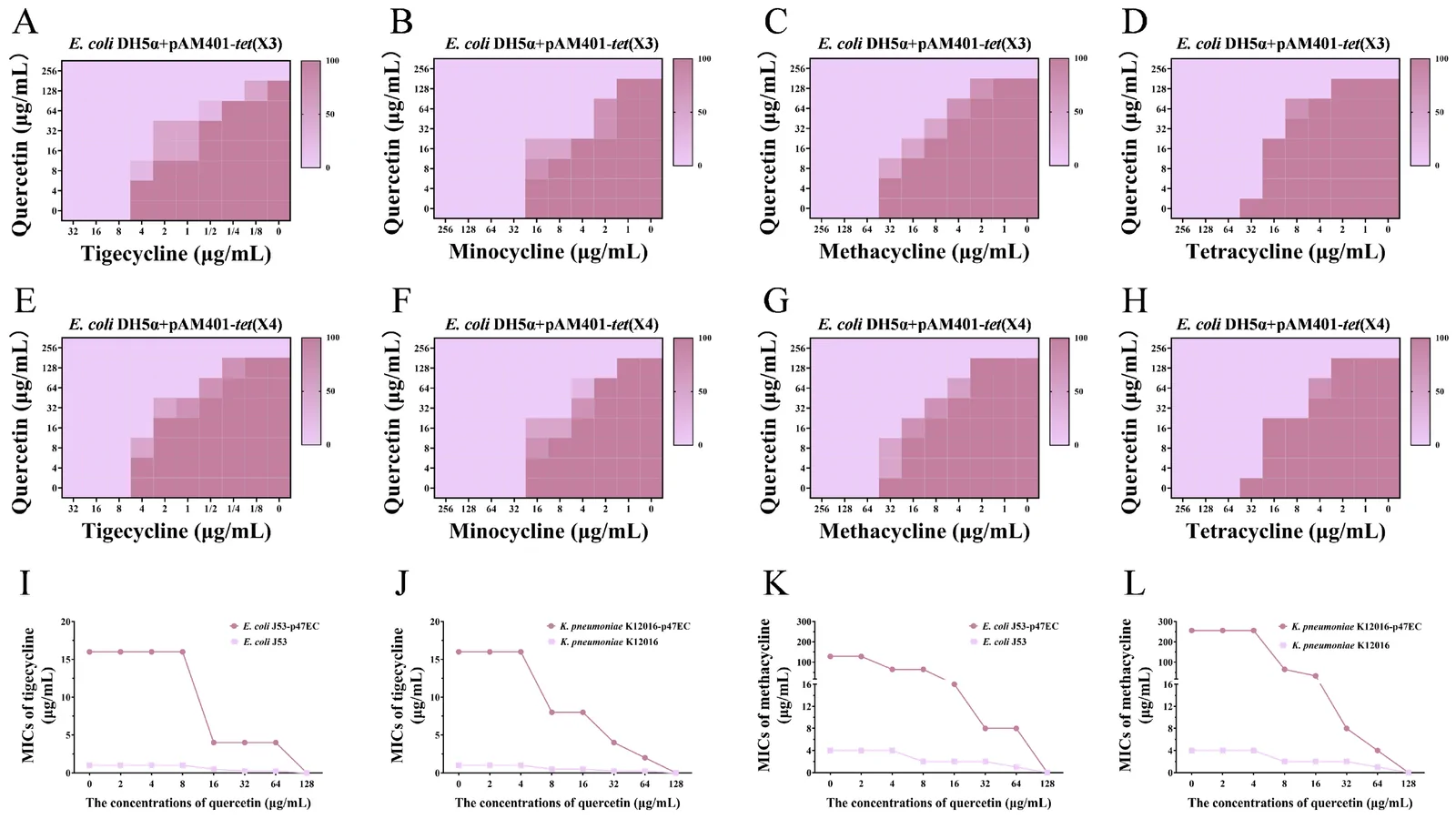
**Antibacterial effects of quercetin in combination with different tetracyclines against Tet(X3)/Tet(X4)-positive bacteria.** The antibacterial effect of quercetin in combination with tigecycline (**A**), minocycline (**B**), methacycline (**C**), or tetracycline (**D**) against *Escherichia coli* DH5α+pAM401-*tet*(X3) was evaluated using the checkerboard minimum inhibitory concentration (MIC) assay. The antibacterial effect of quercetin in combination with tigecycline (**E**), minocycline (**F**), methacycline (**G**), or tetracycline (**H**) against *E. coli* DH5α+pAM401-*tet*(X4) was evaluated using the checkerboard MIC assay. All MICs were assessed in quadruplicate, indicating the likelihood of bacterial proliferation by color variation. (**I**–**L**) The combination of quercetin with tigecycline or methacycline exerted synergistic antibacterial effects against Tet(X4)-positive *E. coli* J53p47EC and *Klebsiella pneumoniae* K12016p47EC and additive antibacterial effects against Tet(X4)-negative *E. coli* J53 and *K. pneumoniae* K12016. Data are represented as mean ± standard deviation.

**Figure 3:**
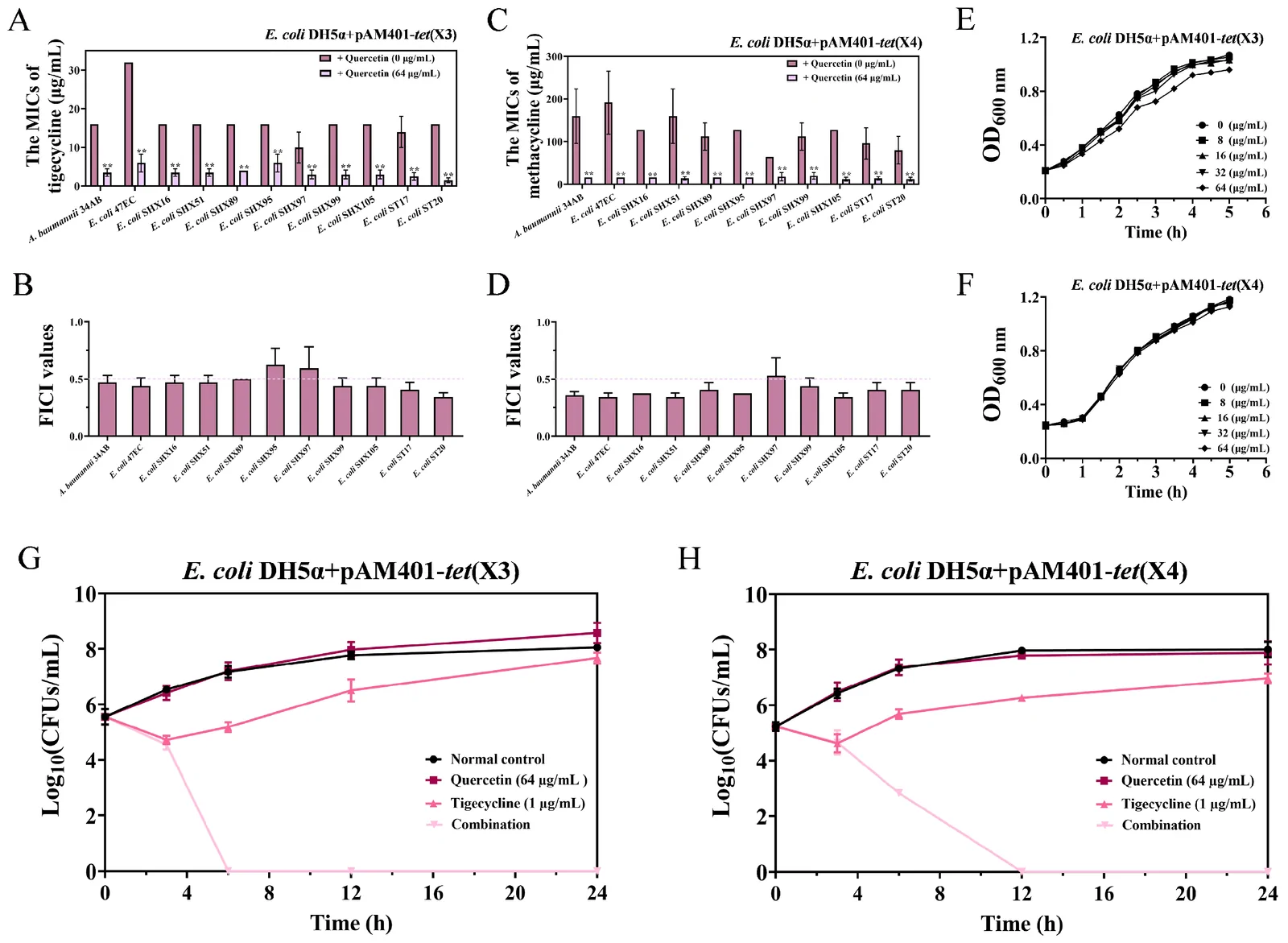
**In vitro synergistic antibacterial activities of quercetin in combination with tetracyclines against Tet(X3)/Tet(X4)-harboring bacterial strains.** (**A**–**D**) The minimum inhibitory concentration (MIC) and fractional inhibitory concentration index (FICI) values of tigecycline/methacycline with or without 64 μg/mL quercetin against 11 different clinical isolates. Growth curves (**E**,**F**) and time-dependent killing assay results (**G**,**H**) for *Escherichia coli* DH5a+pAM401-*tet*(X3) and *E. coli* DH5a+pAM401-*tet*(X4). Data are represented as mean ± standard deviation. ** *p* < 0.01. OD_600nm_, optical density at 600 nm; CFUs, colony-forming units.

The synergistic antibacterial effects of quercetin and different tetracyclines against Tet(X3)-positive and Tet(X4)-positive bacteria were evaluated using the modified checkerboard microdilution method. Quercetin exerted significant synergistic growth-inhibitory effects with tigecycline, minocycline, methacycline, or tetracycline (FICI ≤ 0.5) against *E. coli* DH5α+pAM401-*tet*(X3) (Figure 2A–D) and *E. coli* DH5α+pAM401-*tet*(X4) (Figure 2E–H). A previous study reported that the artificial insertion of *tet*(X4) conferred tetracycline resistance to the clinical isolates *K. pneumoniae* K12016 and *E. coli* J53 [19]. As shown in Figure 2I–L, quercetin significantly and dose-dependently potentiated the antibacterial effect of tigecycline or methacycline against *K. pneumoniae* K12016p47EC and *E. coli* J53p47EC. However, this synergistic effect was not observed against *K. pneumoniae* K12016 and *E. coli* J53, which can be attributed to the absence of *tet*(X4) in the bacterial strains. Quercetin in combination with tigecycline or methacycline also exerted synergistic effects against *tet*(X3)-positive *A. baumannii* 34AB isolates and most *tet*(X4)-positive *E. coli* isolates (including those of chicken origin) and *tet*(X5)-positive *E. coli* ST17 from human (FICI ≤ 0.5) (Figure 3A–D).

**Figure 4:**
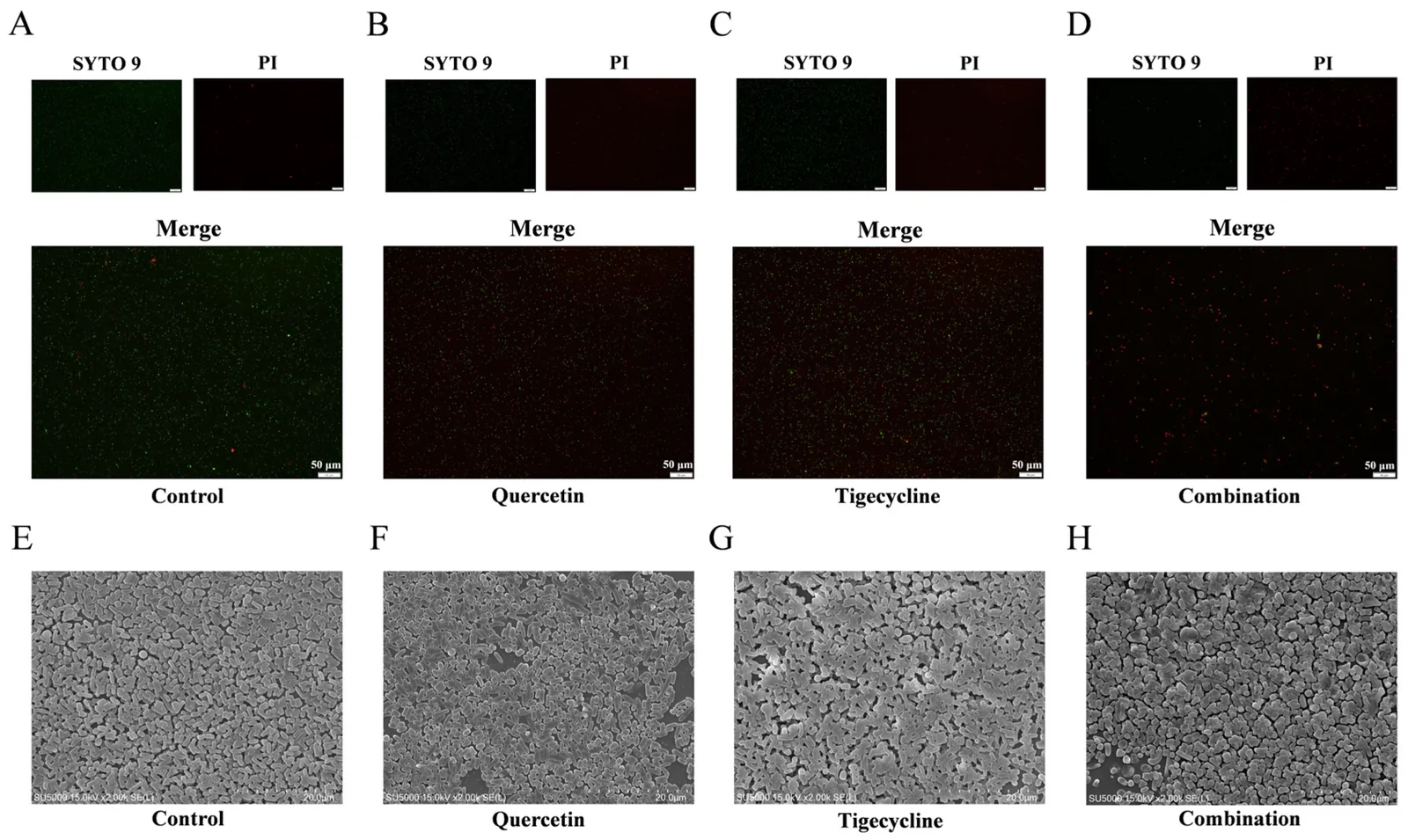
**Morphological observation of the in vitro synergistic antibacterial effects of tigecycline + quercetin.** (**A**–**D**) Fluorescence labeling analysis (scale bar = 50 μm) and (**E**–**H**) scanning electron microscopy (SEM) analysis of *Escherichia coli* DH5a+pAM401-*tet*(X4) in different treatment groups (quercetin (64 μg/mL), tigecycline (1 μg/mL), combination (64 μg/mL quercetin + 1 μg/mL tigecycline), or the control (no treatment)). Scale bar = 20.0 μm. PI, propidium iodide.

**Figure 5:**
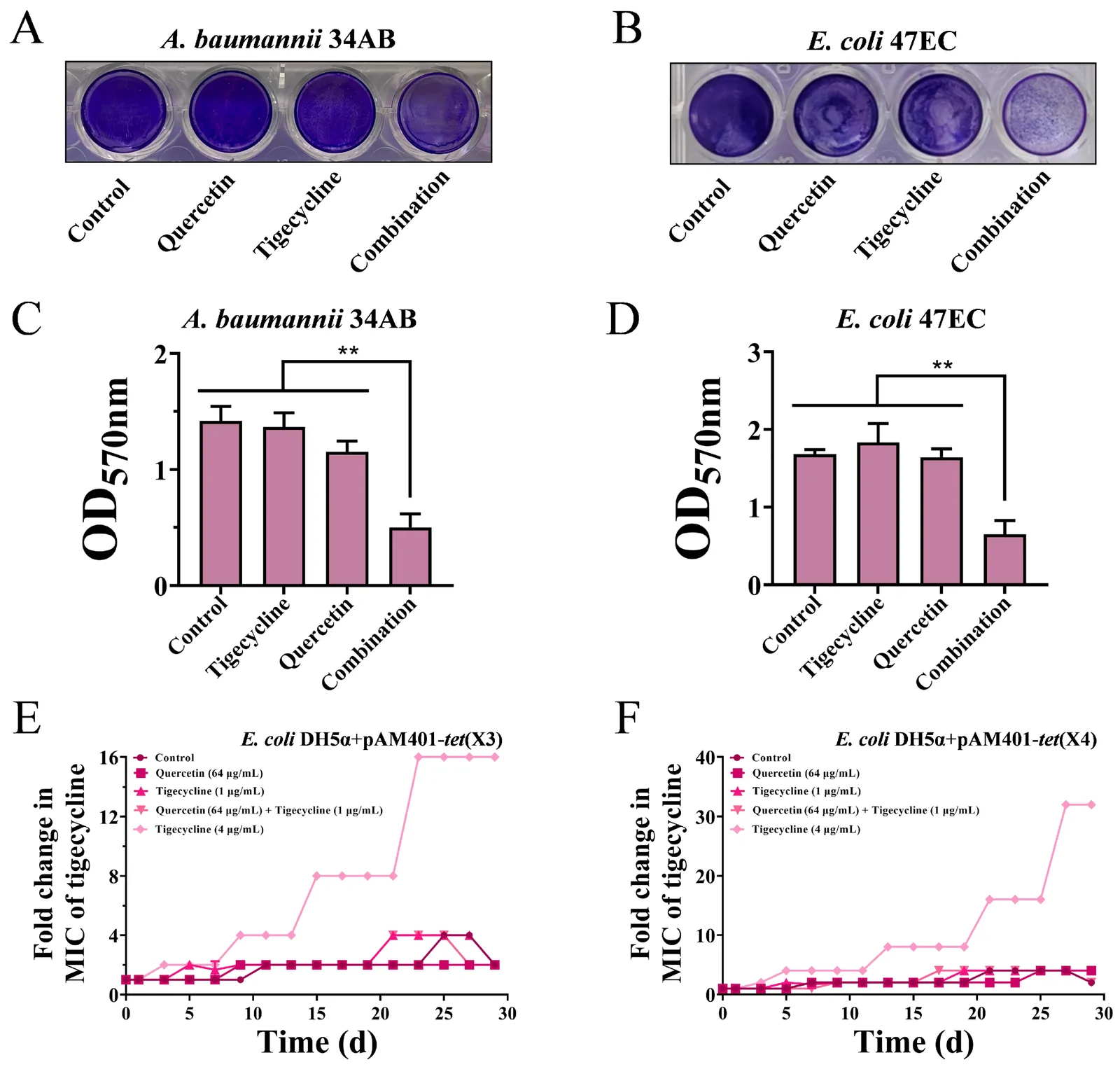
**Quercetin + tigecycline combination significantly reduces the formation of bacterial biofilms.** The biofilm content of *Acinetobacter baumannii* 34AB (**A**,**C**) and *Escherichia coli* 47EC (**B**,**D**) treated with quercetin (64 μg/mL), tigecycline (4 μg/mL), the combination (64 μg/mL quercetin + 4 μg/mL tigecycline), or control (no treatment) was examined using crystal violet (CV) staining (*n* = 3). Treatment with quercetin (64 µg/mL), tigecycline (1 µg/mL), and combination (64 µg/mL quercetin + 1 µg/mL tigecycline) did not induce tetracycline resistance in *E. coli* DH5α+pAM401-*tet*(X3) (**E**) and *E. coli* DH5α+pAM401-*tet*(X4) (**F**) (*n* = 3). All results are represented as the mean ± standard deviation. ** *p* < 0.01. OD_570nm_, optical density at 570 nm; MIC, minimum inhibitory concentration.

The growth curves revealed that quercetin (≤64 µg/mL) did not affect the growth of Tet(X3)-positive *E. coli* DH5α+pAM401-*tet*(X3) and Tet(X4)-positive *E. coli* DH5α+pAM401-*tet*(X4) (Figure 3E,F). Next, the bactericidal effect of the quercetin + tetracycline combination against *E. coli* DH5α+pAM401-*tet*(X3) and *E. coli* DH5α+pAM401-*tet*(X4) was examined in vitro. As shown in Figure 3G,H, the quercetin + tigecycline combination treatment exerted potent bactericidal effects at 6 h (for *E. coli* DH5α+pAM401-*tet*(X3)) or 12 h (for *E. coli* DH5α+pAM401-*tet*(X4)) when compared with the control, quercetin alone, and tigecycline alone treatments. Consistently, integrated analysis using the LIVE/DEAD BacLight bacterial viability kit (red color indicates dead bacteria) (Figure 4A–D) and SEM (Figure 4E–H) revealed that most bacteria in the quercetin alone, tigecycline alone, and control groups exhibited physiological cell morphology. In contrast, bacteria in the quercetin + tigecycline group exhibited markedly small and round-shaped morphology, increased membrane permeability, and an enhanced mortality rate. Additionally, CV staining revealed that quercetin, tigecycline, or quercetin + tigecycline effectively reduced the biofilm biomass of the clinical isolates Tet(X3)-positive *A. baumannii* 34AB and Tet(X4)-positive *E. coli* 47EC. Analysis of the color intensity (Figure 5A,B) and quantitative analysis of the biofilm content (Figure 5C,D) confirmed the antibiofilm activity of the quercetin + tigecycline combination.

The serial passage tigecycline-resistant test demonstrated that *E. coli* DH5α+pAM401-*tet*(X3) and *E. coli* DH5α+pAM401-*tet*(X4) cultured with 4 μg/mL tigecycline exhibited tigecycline resistance when compared with those cultured with 64 μg/mL quercetin, 1 μg/mL tigecycline, 64 μg/mL quercetin + 1 μg/mL tigecycline, and the control (Figure 5E,F). Thus, quercetin effectively restores the bactericidal efficacy of tetracycline in vitro at concentrations that do not compromise bacterial viability or induce tigecycline resistance by inhibiting the activity of Tet(X3)/Tet(X4).

### 3.3. Quercetin Inhibits the Activity of Tet(X4) by Binding to Its Active Center

**Figure 6:**
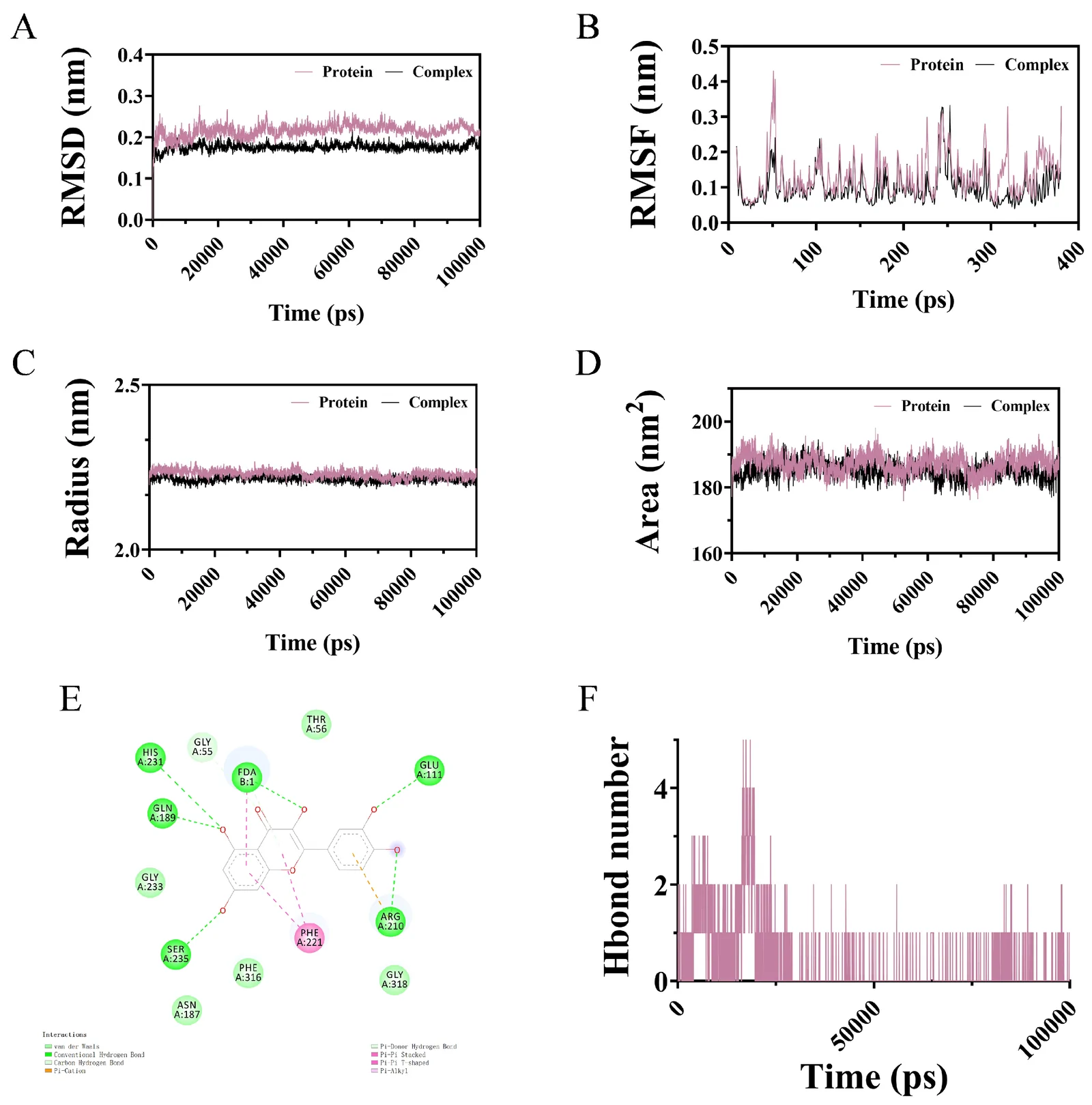
**Critical parameters of molecular dynamics simulation.** The root mean square deviation (RMSD) (**A**), root mean square fluctuation (RMSF) (**B**), radius of gyration (Rg) (**C**), and solvent-accessible surface area (SASA) values (**D**) of Tet(X4) and the Tet(X4)-quercetin complex during molecular dynamics simulation. (**E**) Quercetin binds to Tet(X4) through hydrophobic and hydrophilic interactions. (**F**) The number of hydrogen bonds during the simulation process (0–100,000 ps).

Molecular docking and molecular modeling were performed to investigate the inhibitory effects of quercetin on Tet(X4). After binding to quercetin, the root mean square deviation and the root mean square fluctuation values of the Tet(X4)-quercetin complex were lower than those of the pure Tet(X4) protein system (Figure 6A). Analysis of the radius of gyration (total and around axes) indicated that the binding of quercetin to Tet(X4) increased the structural compactness (Figure 6B). The Tet(X4)-quercetin complex exhibited significantly lower protein residue flexibility than pure Tet(X4). This indicates that quercetin binding stabilizes the protein backbone flexibility (Figure 6C). Furthermore, the pure Tet(X4) and Tet(X4)-quercetin complex exhibited similar levels of hydrophobic/hydrophilic surface exposure in the solvent environment (Figure 6D). These parameters indicate that the MD simulation meets the stability criteria.

**Figure 7:**
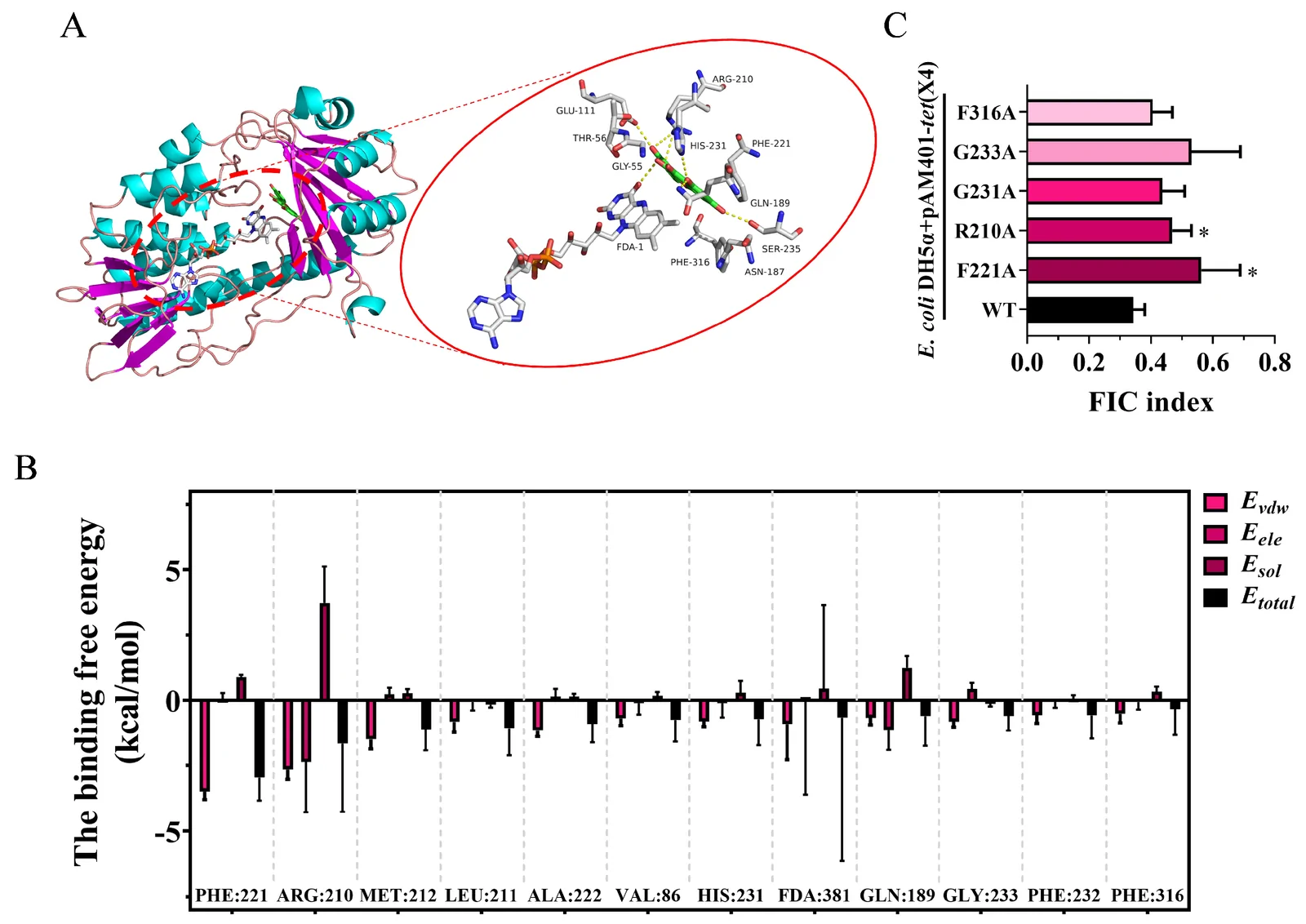
**Quercetin inhibits Tet(X4) activity by directly binding to the active center of Tet(X4).** (**A**) The three-dimensional (3D) structure of the Tet(X4)-quercetin complex. (**B**) Analysis of the binding energy decomposition on a per-residue basis in the Tet(X4)-quercetin complex. (**C**) The synergistic activity of quercetin + tigecycline against *Escherichia coli* DH5a+pAM401-*tet*(X4) was markedly lower than that against wild-type (WT) bacteria. * *p* < 0.05. FIC, fractional inhibitory concentration.

Quercetin occupies an internal cavity within Tet(X4) adjacent to the cofactor FAD, exhibiting favorable shape complementarity, which suggests a strong interaction (Figure 7A). The binding site is characterized by a mixed hydrophobic and hydrophilic environment. Hydrophobic residues, including Phe221 and Phe316, contribute to binding stabilization through pi-alkyl and hydrophobic stacking interactions (Figure 6E). Additionally, quercetin forms key hydrogen bonds with the residues Arg210 and Glu111 and the cofactor FAD, which are critical for binding affinity (Figure 6E,F). Based on MM-GBSA calculations performed over a 50–100 ns trajectory, the computed binding free energy between quercetin and Tet(X4) was Δ*_Etotal_* = −23.43 kcal/mol, indicating a highly favorable and stable interaction (Figure 7B).

To validate the veracity of the molecular docking and molecular modeling outcomes, five mutants of *E. coli* DH5α+pAM401-tet(X4) were constructed. Compared with that against unmutated *E. coli* DH5α+pAM401-tet(X4), the synergistic effect of quercetin and tigecycline decreased against Tet(X4) mutants, although an additive effect was still observed (Figure 7C). The findings suggest that the MD simulation data for the Tet(X4)-quercetin complex are reliable.

### 3.4. Quercetin Potentiates the Protective Effects of Tigecycline Against Tet(X4)-Positive Bacteria in the G. mellonella Larval Infection Model

**Figure 8:**
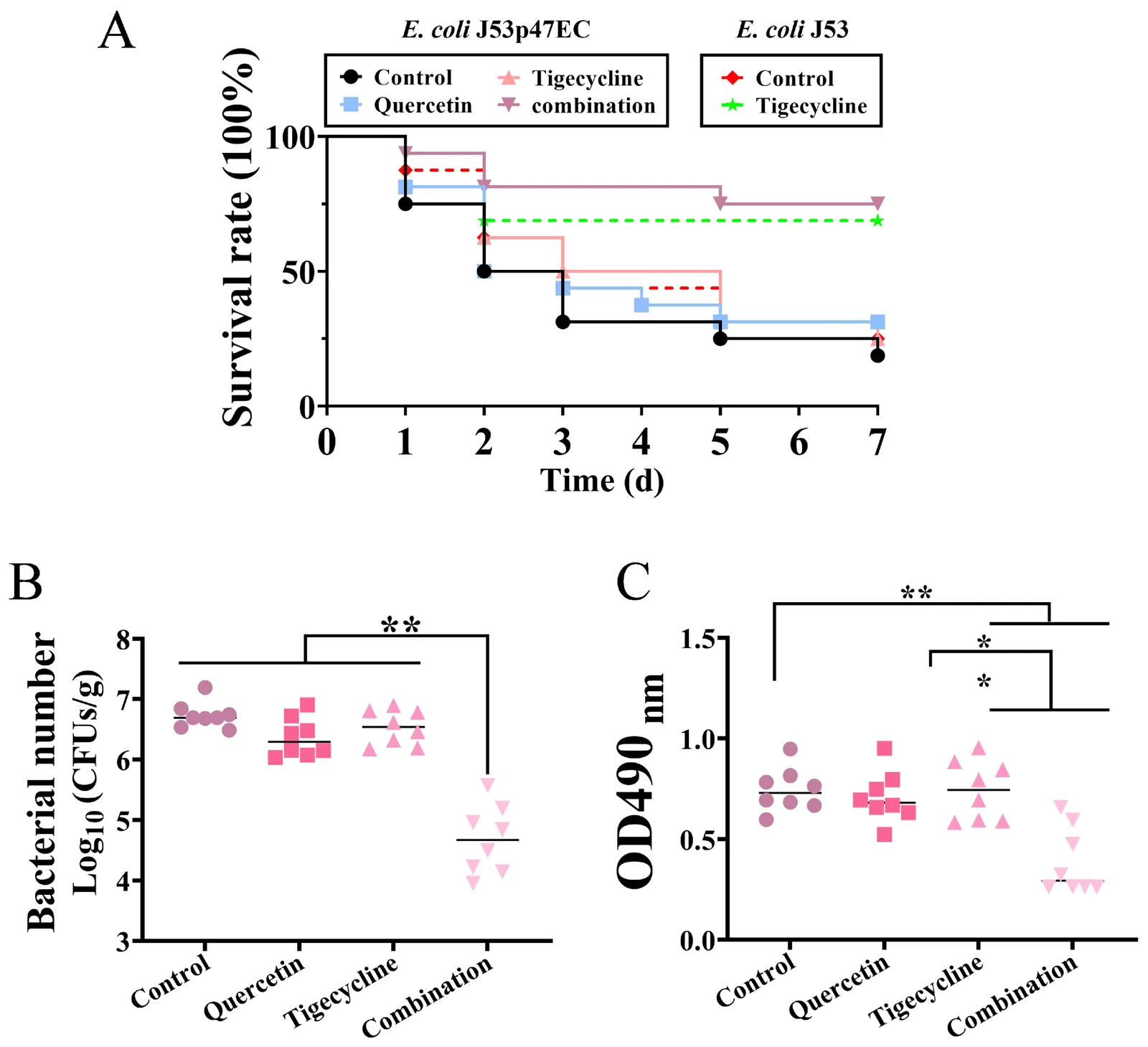
**Synergistic therapeutic effect of quercetin + tigecycline against *Escherichia coli* 47EC in the *Galleria mellonella* larval infection model.** The survival rate (n = 16) (**A**), bacterial load (n = 8) (**B**), and melanization index (n = 8) (**C**) of the *G. mellonella* larval infection model. * *p* < 0.05; ** *p* < 0.01. OD_490nm_, optical density at 490 nm; CFUs, colony-forming units.

This study established an experimental model of *E. coli* infection in the *G. mellonella* larvae to further evaluate the in vivo therapeutic efficacy of the quercetin + tigecycline combination. The therapeutic effect of quercetin + tigecycline was assessed based on the larval survival rates (Figure 8A), bacterial colony counts (Figure 8B), and the melanization index (Figure 8C). Compared with that in the control (18.8%) and tigecycline-treated (25.0%) groups, the larval survival rate was significantly higher in the quercetin + tigecycline group (68.8%) (Figure 8A). Quercetin + tigecycline significantly reduced the bacterial load in the larvae and effectively alleviated pathological damage (Figure 8B,C). Treatment with tigecycline alone was efficacious against Tet(X4)-negative *E. coli* J53 infections (Figure 8A). These findings indicate that Tet(X4) enzymatic inhibition is the predominant pathway through which quercetin enhances the in vivo efficacy of tetracycline antibiotics. The multi-functional biological properties of quercetin may provide auxiliary protective effects in larval hosts. Thus, quercetin serves as a Tet(X4) inhibitor and has potential therapeutic applications for tetracycline-resistant bacterial infections.

## 4. Discussion and Conclusions

The clinical relevance of tetracycline-class antibiotics, which are historically established broad-spectrum antimicrobials, continues to evolve [27,28]. Irrational usage of antibiotics in humans and animals has led to the development of resistance among common pathogens to first-generation, second-generation, and third-generation agents, such as tetracycline, doxycycline, and minocycline [14]. However, the classical resistance mechanisms have not affected the efficacy of tigecycline. According to the CHINET 2023 data, the overall tigecycline resistance rate is low. Thus, tigecycline is a critical therapeutic option for infections caused by carbapenem-resistant *Enterobacteriaceae* and *A. baumannii* [29]. Antibiotic resistance mechanisms involving the plasmid-borne enzymes Tet(X3)/Tet(X4) represent a major threat to the efficacy of tigecycline and other next-generation tetracyclines. The availability of validated inhibitors of Tet(X3)/Tet(X4) is limited. Thus, there is a need to perform high-throughput screening for identifying potent, developable Tet(X3)/Tet(X4) inhibitors to preserve the clinical utility of the tetracycline-class antibiotics [19,20].

Bacterial antibiotic resistance-related enzyme inhibitors, especially β-lactamase inhibitors, have been used in the clinic. This indicates that the development of antibiotic resistance-related enzyme inhibitors is a feasible and pragmatic strategy for combating antimicrobial resistance. Targeting tetracycline resistance-related enzymes offers a mechanism-driven strategy to improve the clinical utility of existing tetracyclines, including next-generation agents, such as eravacycline. These inhibitors exhibit high target specificity and potentially low off-target toxicity, which may attenuate the selective pressure driving collateral resistance. The efficacy of these inhibitors is intrinsically restricted to pathogens harboring cognate resistance determinants, necessitating companion diagnostics. The evolutionary plasticity of bacterial flavoenzymes permits the rapid acquisition of active site mutations—exemplified by the emergence of Tet(X3/4)—or compensatory upregulation. Meanwhile, pharmacokinetic mismatches between inhibitor and partner tetracycline can compromise synergistic exposure, constraining translational viability.

The antioxidant activity of quercetin, a naturally occurring flavonol (C_15_H_10_O_7_; 302.23 g/mol), can be attributed to its polyphenolic scaffold. Quercetin is predominantly isolated from dietary sources, including onion scales, apple peels, and blueberries [30,31]. Additionally, quercetin often exists in the form of glycosides (such as rutin) and undergoes enzymatic hydrolysis in vivo to release the bioactive aglycone [30,31]. Quercetin activates the Nrf2/ARE signaling axis, upregulating endogenous antioxidant enzymes (including superoxide dismutase and glutathione peroxidase) and suppressing pro-inflammatory pathways by selectively inhibiting COX-2 and NF-κB, which results in the suppression of the production of TNF-α and IL-6 [32,33,34]. The antitumor efficacy of quercetin can be attributed to the induction of cell cycle arrest and mitochondrial-dependent apoptosis and the inhibition of VEGF-driven angiogenesis. Previous studies have demonstrated that quercetin exerts cytostatic effects against breast and colorectal cancer cell lines in vitro [35,36,37]. Quercetin has been employed in managing chronic bronchitis, hypertension, and coronary heart disease. However, the therapeutic translation of quercetin is constrained by poor oral bioavailability and rapid systemic clearance [31,35]. To overcome these pharmacokinetic limitations, efforts are ongoing to develop nanoformulations and delivery systems. The discovery of quercetin as a direct inhibitor of Tet(X3)/Tet(X4) significantly expands its pharmacological repertoire and its potential for development as a clinical drug [35].

The duration for developing a novel antimicrobial agent is more than a decade. However, bacterial resistance can emerge within one to two years. Consequently, the identification of novel resistance enzyme inhibitors has piqued the interest of the scientific community. Several previously identified inhibitors have potential for clinical translation. This study comprehensively evaluated the synergistic antibacterial activity of quercetin + tetracyclines against clinical and laboratory strains harboring *tet*(*X3*) or *tet*(*X4*). Furthermore, the potential mechanisms through which quercetin directly targets the catalytic center of Tet(X4) to inhibit its enzymatic function were elucidated. Finally, the feasibility of this combinatorial strategy was demonstrated in vivo using a *G. mellonella* larval infection model challenged with Tet(X4)-positive pathogens.

Cytotoxicity and hemolysis assay results revealed that the effective synergistic concentration of quercetin exerted negligible toxic effects on mammalian lung epithelial cells, macrophages, and rabbit erythrocytes. Additionally, the combination of quercetin and tigecycline did not aggravate host cell damage. Future studies should focus on optimizing quercetin nano-delivery formulations to improve its systemic bioavailability, performing mammalian animal model efficacy verification, developing quercetin structural derivatives with potent Tet(X) inhibitory activity, and conducting long-term serial passage resistance induction tests and whole-genome sequencing to comprehensively evaluate the risk of resistance evolution under combination treatment.

## Data Availability

The data are available from the corresponding authors upon reasonable request.
